# A risk‐organised model for clinically significant prostate cancer early detection

**DOI:** 10.1002/bco2.230

**Published:** 2023-03-07

**Authors:** Juan Morote, Ángel Borque‐Fernando, Marina Triquell, José M. Abascal, Pol Servian, Jacques Planas, Olga Mendez, Luis M. Esteban, Enrique Tilla

**Affiliations:** ^1^ Department of Urology Vall d'Hebron Hospital Barcelona Spain; ^2^ Department of Surgery Universitat Autònoma de Barcelona Barcelona Spain; ^3^ Department of Urology Hospital Miguel Servet, IIS‐Aragon Zaragoza Spain; ^4^ Department of Urology Parc de Salut Mar Barcelona Spain; ^5^ Department of Surgery Universitat Pompeu Fabra Barcelona Spain; ^6^ Department of Urology Hospital Germans Trias i Pujol Badalona Spain; ^7^ Urology Biomedical Research Unit Vall d'Hebron Research Institute Barcelona Spain; ^8^ Department of Applied Mathematics, Escuela Universitaria Politécnica La Almunia Universidad de Zaragoza Zaragoza Spain

**Keywords:** clinically significant, mpMRI, prostate cancer, risk‐organised model

Evidence that specific mortality of PCa decrease when clinically significant PCa (csPCa) is early detected has moved the focus of PCa screening towards csPCa.^1^ This paradigm shift has occurred since the spread of multiparametric magnetic resonance imaging (mpMRI), which allows to avoid unnecessary prostate biopsies and overdetection of insignificant PCa (iPCa) in a cost‐effective way.^2^, ^3^ However, because suspicion of PCa remains based on elevated serum prostate‐specific antigen (PSA) and/or abnormal digital rectal examination (DRE), there has been an increased demand for mpMRI that cannot always be performed. In experienced centres, biparametric MR has replaced mpMRI, reducing scan time by a quarter and maintaining the reproducibility and accuracy of the Prostate Imaging Reporting and Data System (PI‐RADS).^1^

The recommendation of prostate biopsy is currently made according to the PI‐RADS category. Experienced radiologists, reporting with an updated version of PI‐RADS, obtain a negative predictive value of mpMRI that reaches up to 95%, which makes it possible to avoid prostate biopsies in men with suspected PCa with a PI‐RADS <3. MRI‐targeted biopsies of suspicious lesions (PI‐RADS ≥3) improve the sensitivity of systematic biopsies for csPCa. However, uncertain scenarios after mpMRI, having high rates of unnecessary biopsies and/or overdetection of iPCa, remain, and then PSA density (PSAD), new markers and predictive models are recommended to improve the selection of candidates for prostate biopsy.^4^ The European Association of Urology currently recommends the design of csPCa risk‐organised models (ROMs) by sequencing available tools to reduce the demand of mpMRI exams, and unnecessary prostate biopsies ones mpMRI is performed.^1^ Because prostate volume is a powerful predictor of csPCa and usually transrectal ultrasound is not performed to assess prostate volume before mpMRI, its assessment through DRE‐prostate volume category is now recommended.^5^ There is also evidence that men with serum PSA higher than 10 ng/mL and abnormal DRE do not benefit from MRI‐targeted biopsies, since systematic biopsies can detect all existing csPCa.^6^ The Barcelona‐risk calculator 1 (BCN RC‐1) has been developed and externally validated to individualise the risk of csPCa to avoid the demand of mpMRI exams,^7^ as well as the BCN‐RC 2 to predict the risk of csPCa after mpMRI and avoid unnecessary biopsies.^7^ Both risk calculators are available at https://mripcaprediction.shinyapps.io/MRIPCaPrediction/.

The present study aims to compare the current standard approach for early detection of csPCa, based on MRI‐targeted biopsies when PI‐RADS lesions ≥3 and systematic biopsy,^1^ with a ROM designed to avoiding mpMRI exams in men with serum PSA over than 10.0 ng/mL and abnormal DRE, in addition to rule out mpMRI exams when the risk of csPCa from the BCN‐RC 1 is lower than 12%.^6^, ^7^ Once mpMRI is performed, prostate biopsy will be scheduled in men having a risk of csPCa from the BCN‐RC 2 higher than 4%.^8^ The selection of proposed thresholds was made to avoid missing no more than 10% of csPCa detected. The 95% csPCa sensitivity thresholds of BCN RC‐1 and BCN RC‐2, in those men in whom they were applied, were selected. A probability analysis of avoiding mpMRI exams and prostate biopsies as well as missed csPCa has been performed.

A series of 946 men with serum PSA > 3.0 ng/mL and/or abnormal DRE was recruited prospectively in two academic centres of cities from the Barcelona metropolitan area (PSM and GTiP), between January 1 of 2018 and December 31 of 2021. This series was independent from those for the development of BCN‐RC 1 and BCN‐RC 2 recruited at VHH, between January 1 of 2016 and December 31 of 2019.^7^, ^8^ All men were scheduled to 3‐T mpMRI and two‐ to four‐core transrectal ultrasound (TRUS) cognitive MRI‐targeted biopsies to PI‐RADSv.2 ≥ 3 lesions and 12‐core TRUS systematic biopsy; 12‐core TRUS systematic biopsy was performed when PI‐RADSv.2 < 3. This project was approved by the institutional ethics committee of VHH (PRAG‐317/2017), and the analysis was performed on anonymised databases. CsPCa, defined as the International Society of Uro‐pathology grade group 2 or higher, was detected in 386 men (40.8%). The median age of participants was 67 years with an interquartile range (IQR) between 61 and 75. The median serum PSA was 7.2 ng/mL (IQR: 5.5–10.9), 32.5% of participants had abnormal DRE, and 31% had previous negative prostate biopsy. CsPCa was detected in 17.9% of the 235 men with PI‐RADS <3 (24.8%); in 20.4% of the 301 men with PI‐RADS 3 (21.2%); in 51.9% of the men with PI‐RADS 4 (12.6%); and 84% of those with PI‐RADS 5 (12.6%). In the subset of 124 men with serum PSA > 10.0 ng/mL and abnormal DRE, csPCa was detected in 106 (85.6%).

The probability analyses of the standard approach and the proposed ROM are presented in Figure 1. The standard approach required mpMRI in all participants, 235 (24.8%) of prostate biopsies were avoided in those men with PI‐RADS <3, and 42 (10.9%) of overall csPCa detected were missed. Among the 711 participants biopsied (75.2%) csPCa was detected in 344 (48.4%). The proposed ROM initially ruled out mpMRI in 124 men with serum PSA > 10.0 ng/mL and abnormal DRE (13.1% of all participants), in whom systematic biopsies identified all 106 csPCa detected, which represented 27.5% of all csPCa detected. The BCN‐RC 1 ruled out mpMRI exams in 167 men (17.7%), missing 13 csPCa (3.4%). After mpMRI, performed in 655 men (69.2%), the BCN RC‐2 ruled out prostate biopsy in 100 men, in whom 13 detected csPCa were missed (3.4%). Among the 555 men finally biopsied (63.3%), csPCa was detected in 254 (45.8%). The ROM would rule out 30.8% of mpMRI exams, and 28.2% of prostate biopsies, whereas 6.7% of overall csPCa would be undetected.

**FIGURE 1:**
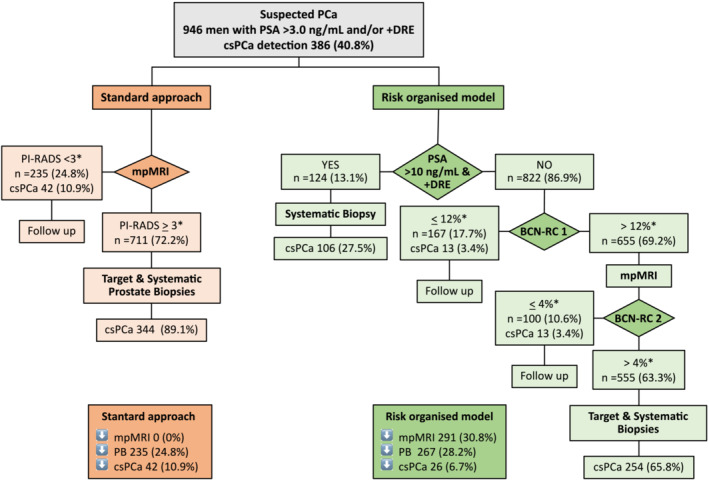
Flow chart description of standard approach of csPCa, and the proposed risk‐organised model, with intermediate and overall results according to rule out mpMRI exams, avoided prostate biopsies and missed csPCa detection. Data are expressed in number and (%) of all MRI exams and prostate biopsies performed and csPCa detected. Abbreviations: BCN, Barcelona; csPCa, clinically significant PCa; DRE, digital rectal examination; mpMRI, multiparametric magnetic resonance imaging; PB, prostate biopsies; PCa, prostate cancer; PI‐RADS, Prostate Imaging Report and Data System; PSA, prostate‐specific antigen; RC, risk calculator. ^a^Proposed thresholds.

The proposed ROM was able to rule out almost one‐third of mpMRI exams and the percentage of saved biopsies increased from the 24.8% observed with the standard approach to the 28.2%, whereas the percentage of undetected csPCa decreased from 10.9 to 6.7 respectively. Remmers et al. have recently reported the results with an ROM based on sequencing the Rotterdam RC‐3 and the Rotterdam MRI‐RC in the MRI arm of the PRECISION trial, which was carried out in biopsy‐naïve men. After recalibration and adjustment of csPCa thresholds in both predictive models, this ROM was able to rule out 13% of mpMRI exams, decreasing the number of prostate biopsies in 9% and missing 8.5% of csPCa detected in the 134 men with PI‐RADS ≥3 in whom MRI‐targeted biopsies of suspicious lesions and systematic biopsy were performed.^9^

The present study confirms the effectiveness of the proposed ROM to improve the early detection of csPCa by reducing the demand of mpMRI exams and unnecessary prostate biopsies. The missing rate of csPCa of the standard approach that avoids systematic biopsy in men with negative mpMRI also decreased. The main limitation for the use of the proposed ROM is the need of validation in the populations where it will be implemented. Now, following the recommendation of EAU, this ROM is ready to be used in the metropolitan area of Barcelona, and validation in Catalonia (Spain), a country with seven and half million inhabitants, is ongoing. Ideally, a randomised trial would be necessary to generate high evidence level.

## AUTHOR CONTRIBUTIONS

Juan Morote, Ángel Borque‐Fernando and Luis M. Esteban conceptualised the idea. Marina Triquell, José M. Abascal, Pol Servian, Jacques Planas, Olga Mendez and Enrique Tilla developed the concept. Juan Morote wrote the first draft of the manuscript. All authors were involved in editing, critical review and final approval of the manuscript.

## CONFLICT OF INTEREST STATEMENT

The authors have no conflict of interest to declare.
